# Hypoxia activates Wnt/β-catenin signaling by regulating the expression of BCL9 in human hepatocellular carcinoma

**DOI:** 10.1038/srep40446

**Published:** 2017-01-11

**Authors:** Wei Xu, Wang Zhou, Mo Cheng, Jing Wang, Zhian Liu, Shaohui He, Xiangji Luo, Wending Huang, Tianrui Chen, Wangjun Yan, Jianru Xiao

**Affiliations:** 1 Department of Orthorpedic Oncology, Changzheng Hospital, Second Military Medical University, NO. 415, Fengyang Road, Shanghai, 200003, China; 2 Department of Bone and soft tissue tumors, Fudan Cancer Center, Fudan University, NO. 270, Dong’an Road, Shanghai, 200000, China; 3 Department of Anatomy, Xuzhou Medical University, NO. 209, Tongshan Road, Xuzhou, 221004, China; 4 Department of Biliary Surgery, Eastern Hepatobiliary Surgery Hospital, Second Military Medical University, NO. 225, Changhai Road, Shanghai, 200438, China

## Abstract

The Wnt/β-catenin signaling is abnormally activated in the progression of hepatocellular carcinoma (HCC). BCL9 is an essential co-activator in the Wnt/β-catenin signaling. Importantly, BCL9 is absent from tumors originating from normal cellular counterparts and overexpressed in many cancers including HCC. But the mechanism for BCL9 overexpression remains unknown. Ample evidence indicates that hypoxia inducible factors (HIFs) play a role in the development of HCC. It was found in our study that BCL9 was overexpressed in both primary HCC and bone metastasis specimens; loss of BCL9 inhibited the proliferation, migration and angiogenesis of HCC; and that that hypoxia mechanically induced the expression of BCL9. BCL9 induction under the hypoxic condition was predominantly mediated by HIF-1α but not HIF2α. *In vitro* evidence from xenograft models indicated that BCL9 promoter/gene knockout inhibited HCC tumor growth and angiogenesis. Notably, we found that BCL9 and HIF-1α were coordinately regulated in human HCC specimen. The above findings suggest that hypoxia may promote the expression of BCL9 and associate with the development of HCC. Specific regulation of BCL9 expression by HIF-1α may prove to be an underlying crosstalk between Wnt/β-catenin signaling and hypoxia signaling pathways.

Hepatocellular carcinoma (HCC) is the third leading cause of cancer-related mortality worldwide^1^. Despite the increased knowledge about the molecular pathogenesis of HCC and unveiling of promising new therapies, the prognosis of HCC patients remains extremely poor. Therefore, continual efforts are required to develop novel and more effective therapies for the treatment of HCC.

The canonical Wnt/β-catenin signaling pathway is known to be essential for tumorigenesis, and abnormally activated in the progression of HCC^2^,^3^. β-catenin is reported to play a key role in this pathway. In the absence of Wnt ligands, β-catenin is phosphorylated and degraded by the destruction complex consisting of adenomatous polyposis coli (APC), Axin, glycogen synthase kinase-3β (GSK3β), and casein kinase 1α^4^,^5^, while in the presence of Wnt ligands, this destruction complex is dissociated and the unphosphorylated active β-catenin continuously accumulates and translocates to the nucleus. Nuclear β-catenin functions as a transcription factor to activate the expression of cell proliferation, migration, and survival genes such as c-MYC and CyclinD1^6^,^7^. Besides, this transcription pathway can also be activated by a variety of loss-of-function mutations in APC and Axin, as well as by activating mutation in β-catenin itself. These mutations make β-catenin escape degradation and promote the oncogenic transcription.

β-catenin mediated transcription requires several co-activators, including Pygopus (PYGO), B-cell lymphoma 9 (BCL9), and its homologue B-cell lymphoma 9-like (B9L), among others^8^,^9^,^10^. BCL9 is an essential co-activator in the Wnt/β-catenin signaling pathway by mediating the recruitment of pygopus to the nuclear β-catenin-TCF complex^8^. Efficient β-catenin-mediated transcription is required in mammalian cells^11^. In addition, BCL9 enhances β-catenin-mediated transcription activity regardless of the mutational status of the Wnt signaling components and increases cell proliferation, invasion and migration. Importantly, BCL9 is absent from the normal cellular counterparts from which tumors originate^12^. BCL9 is frequently overexpressed in a variety of solid tumors including colorectal cancer, multiple myeloma and HCC. Overexpression of BCL9 is associated with poor prognosis of HCC patients^13^. There is evidence that BCL9 is a bona fide oncogene^12^,^14^,^15^. However, the mechanism of BCL9 overexpression in tumors remains unclear.

Hypoxia is a common feature of all solid tumors and plays an essential role in tumor occurrence and development^16^. The hypoxia microenvironment could be found in HCC because of imbalance between oxygen supply and consumption in proliferating tumors^17^. Ample evidence indicates that hypoxia-inducible factors (HIFs) play an important role in the pathogenesis and pathophysiology of HCC^18^. Thus, HIF inhibitors have been considered as promising drug targets to be exploited in oncology^19^,^20^. Human HIFs are heterodimeric transcription factors consisting of a constitutively expressed subunit (ARNT) and an oxygen-regulated subunit, mainly HIF1α and HIF2α. They promote adaptation of tumor cells to hypoxic stress by regulating the expression of genes involved in metabolism, angiogenesis, cell proliferation and apoptosis^21^,^22^,^23^,^24^. Both *in vitro* and *in vivo* experiments have demonstrated the existence of a crosstalk between the Wnt/β-catenin and HIF pathways^25^. An interaction was found between β-catenin and HIF-1α, implying an underlying competition for β-catenin between HIF-1α and T-cell factor-4^26^. Given the complex mechanism underlying this crosstalk, further efforts should be made to investigate the network of Wnt/β-catenin and hypoxia signaling pathways^24^,^25^,^26^,^27^.

In this study, we studied the effect of hypoxia on BCL9 expression in HCC cells. We firsty found that BCL9 was transcriptionally induced by hypoxia in HCC cell lines in a HIF1α-dependent manner. HIF1α and BCL9 were coordinately regulated in HCC. We demonstrated that the mechanism of regulation on BCL9 was through the hypoxia signaling pathway, thus providing convincing evidence that BCL9 functions as a crucial molecule in hypoxia modulation of Wnt/β-catenin and plays a vital role in carcinogenesis of human HCC.

## Results

### Overexpression of BCL9 protein in human HCC specimens

**Figure 1:**
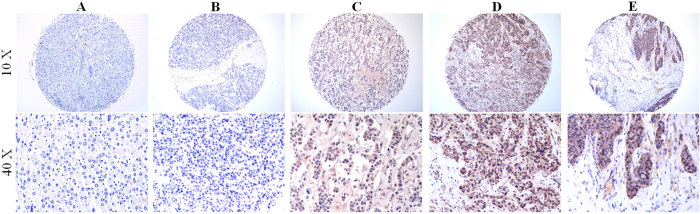
BCL9 expression in primary and metastastic HCC tissue. Representative immunohistochemical stained imaging of BCL9 expression in different developing stages of HCC tissue containing 30 normal liver tissues, 360 primary HCC tissues and 72 bone metastatic HCC tissues. (**A**) No BCL9 staining in the normal mucosal epithelium; **(B)** low intensity expression in primary HCC tissue scored as negative (−); **(C)** moderate intensity expression in primary HCC tissue scored as weak (+); **(D)** high intensity expression in primary HCC tissue scored as strong (++); **(E)** BCL9 staining in bone metastatic HCC tissues. Scale bar: 40 μm for low magnitude image (10×); 10 μm for high magnitude images (40×).

Immunohistochemical studies on primary HCC have demonstrated that BCL9 overexpression is associated with poor prognosis of HCC patients^13^. To further confirm and evaluate the prognostic significance of BCL9 in HCC, we detected the protein expression of BCL9 in the normal liver, primary HCC and bone metastatic specimens by immunohistochemical staining. The sections were scored as negative (−), weak (+) and strong (++) in cases examined. Different distributions of stained tumor cells are shown in Fig. 1. The specimens comprised 30 normal liver tissues, 360 primary HCC tissues and 72 bone metastatic tissues (Table 1). It was found that the expression of BCL9 was significantly increased in HCC specimens, especially in bone metastatic specimens, as compared with that in the normal liver specimens (34.4% and 90.3% *vs.* 0.00%, *P* = 0.00). The correlations between BCL9 expression and clinicopathological parameters in primary HCC are shown in Supplementary Table 1. The expression of BCL9 was correlated with Edmondson invasion (*P* = 0.00), Microvascular invasion (*P* = 0.01) and T-stage (*P* = 0.02). No significant correlation was observed between BCL9 expression and age (*P* = 0.09), gender (*P* = 0.10), tumor size (*P* = 0.92), lymph node metastasis (*P* = 0.15) and distance metastasis (*P* = 0.26). These data together indicate that BCL9 was closely associated with the prognosis of human HCC.

### BCL9 promotes cell proliferation, migration and angiogenesis

**Figure 2:**
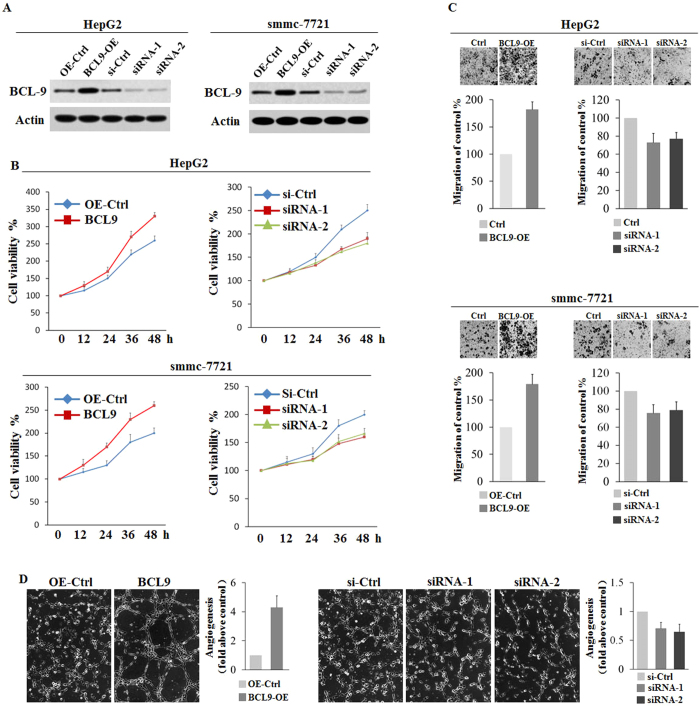
BCL9 promotes cell proliferation, migration, and angiogenesis. (**A**) Over-expression and knockdown BCL9 in HepG2 and smmc-7721 cell lines. **(B)** BCL9 over-expression significantly increases cell viability, while BCL9 knockdown reduces cell viability in HepG2 and smmc-7721 cell lines. **(C)** BCL9 over-expression significantly increases the cell migration ability, while BCL9 knockdown reduces the cell migration ability in HepG2 and smmc-7721 cell lines. **(D)** BCL9 over-expression significantly increases the angiogenesis activity, while BCL9 knockdown reduces the angiogenesis activity in HepG2 cell line. Data are presented as mean ± SD (*n* = 3). *: *p* < 0.01, Student’s *t*-test.

To evaluate the role of BCL9 in human HCC, we used gain-of-function and loss-of-function approaches to evaluate the function of BCL9 in human liver cancer cell lines. Two distinct BCL9 siRNAs were used to avoid the off-target RNA interference effect. (Fig. 2A) Our results suggest that transient BCL9 over-expression significantly increased the cell viability, cell migration and angiogenesis of HepG2 and smmc-7721 cell lines, while BCL9 knockdown reduced the cell viability, migration and angiogenesis of these cell lines. (Figure 2B,C and D) These results suggest that BCL9 was an essential protein for the proliferation, migration and angiogenesis of human HCC cells.

### BCL9 functions as a crucial molecule in hypoxia activation of Wnt/β-catenin pathway

**Figure 3:**
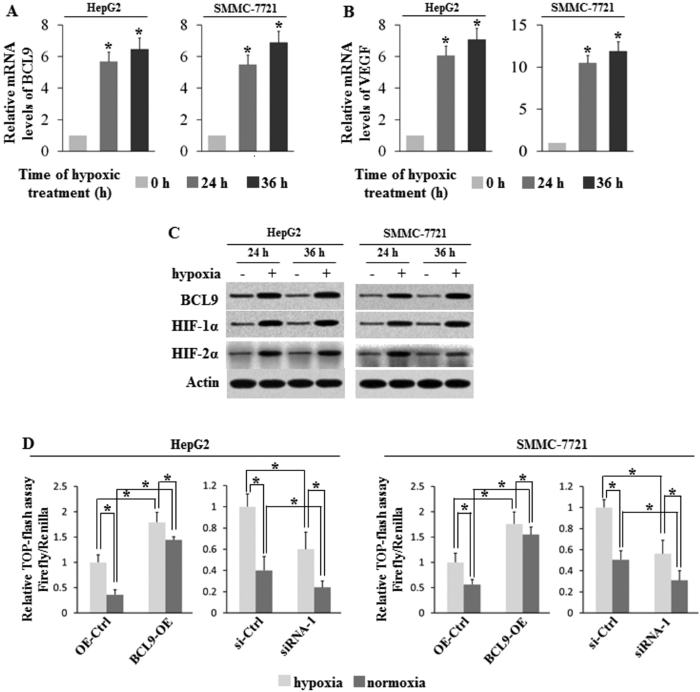
Hypoxia induces BCL9 expression in HCC cells. Human HCC cell lines HepG2 and SMMC-7721 cells were cultured under the hypoxic condition for the indicated time periods. **(A)** The mRNA expression levels of BCL9 in these cells were determined by Taqman real-time PCR and normalized with actin. **(B)** The mRNA expression levels of VEGF in these cells were determined as a positive control. **(C)** The BCL9, HIF-1α and HIF-2α protein levels were determined by Western-blot assays. **(D)** Significant increase in TOPflash activity was observed under hypoxia treatment. Further, the TOPflash activity was significantly increased when BCL9 overexpressed while decreased when BCL9 knocked down. Data are presented as mean ± SD (n = 3).*p < 0.01, Student’s *t*-test.

The previous results showed that BCL9 was overexpressed in primary HCC and bone metastatic specimens, but the underlying mechanism was unclear. Knowing that hypoxia is a common characteristic of the microenvironment of solid tumors including HCC^18^,^28^, we subjected HepG2 and smmc-7721 cells to hypoxia treatment (0.1% O_2_) to see whether hypoxia could modulate the expression of BCL9 in human HCC. The effective induction of hypoxia in cells was confirmed by the increased expression of vascular endothelial growth factor (VEGF), a hypoxia-responsive gene, at mRNA levels (Fig. 3B) and the increased protein levels of HIF-1α and HIF-2α, two major HIFs (Fig. 3C). Interestingly, we found that BCL9 expression was induced by hypoxia (Fig. 3A and C). The results showed that BCL9 mRNA and protein levels were significantly increased in both HepG2 and smmc-7721 cell lines cultured under the hypoxic condition, indicating that the expression of BCL9 was modulated by hypoxia. To test whether hypoxia activated the WNT/β-catenin pathway, a TOPflash luciferase assay was performed. A significant increase in TOPflash activity was observed under hypoxia treatment. (Figure 3D) In addition, the TOPflash activity was significantly increased when BCL9 was overexpressed, and decreased when BCL9 was knocked down. (Figure 3D) These results suggest that BCL9 may function as a crucial molecule in hypoxia activation of the Wnt/β-catenin pathway.

### HIF1α rather than HIF2α binds to HRE-B and HRE-C in BCL9 promoter-induced BCL9 expression under the hypoxia condition

**Figure 4:**
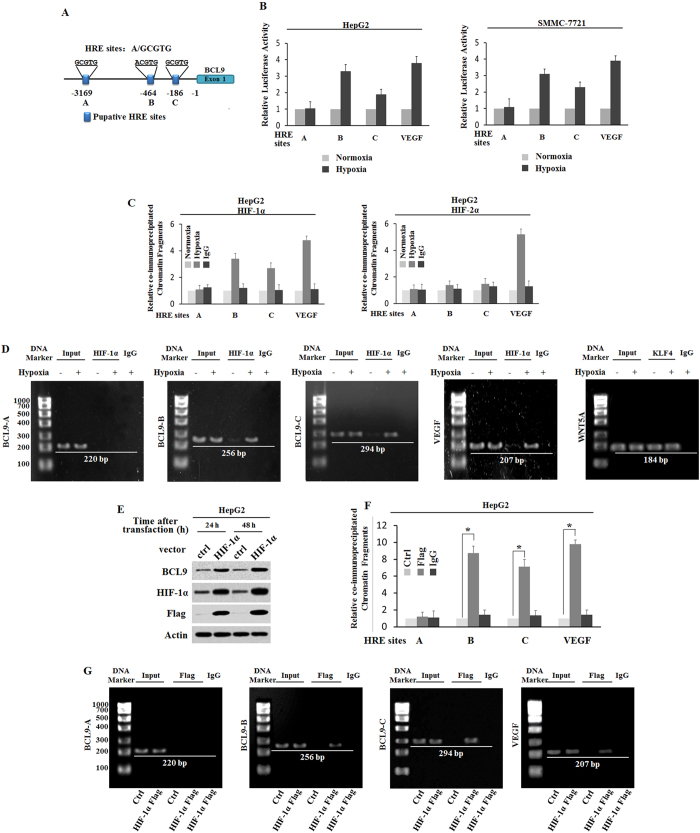
Hypoxia transactivates hypoxia-responsive elements (HREs) in the BCL9 promoter which transcriptionally regulated by HIF-1α. (**A**) The human BCL9 gene contains 3 putative HREs in its promoter region. (**B**) Hypoxia activates the luciferase activity of reporter vectors containing HRE-B or HRE-C sites in the BCL9 promoter. HepG2 and SMMC-7721 cells were transfected with the luciferase reporter vectors, and then subjected to hypoxia treatment for 36 h before measuring luciferase activities. Luciferase reporter vectors containing the HRE site in the VEGF promoter was included as a positive control. (**C,D**) HIF-1α but not HIF-2α binds to HRE-B and HRE-C sites in the BCL9 promoter under the hypoxic condition in HepG2 cells as determined by ChIP assays. Cells were cultured under the hypoxic or normoxic conditions for 36 h before assays. The HRE site in the VEGF promoter serves as a positive control. The amount of DNA fragments pulled- down was determined by real-time PCR (**C**) or conventional PCR (**D**). (**E**) Ectopic HIF-1α expression increases BCL9 protein levels in HepG2 cells as determined by Western-blot assays. (**F,G**) HIF-1α binds to HRE-B and HRE-C sites in the BCL9 promoter in HepG2 cells transfected with HIF-1α expression plasmids as determined by ChIP assays. The amount of DNA fragments pulled-down was determined by real-time PCR (**F**) or conventional PCR (**F**). The HRE site in the VEGF promoter serves as a positive control. Data are presented as mean ± SD (n = 3). *p < 0.01 (Student’s *t*-test).

The transcriptional response to hypoxia in cells is largely mediated by HIFs. Although HIF1a and HIF2a have shared targets such as VEGF, they also regulate unique gene targets^29^,^30^. To study whether the transcriptional induction of BCL9 by hypoxia was mediated by HIFs, we searched for the hypoxia-responsive element (HRE) consensus sequence in the promoter region of the BCL9 gene from 3 kb upstream of transcriptional site to exon 1. Three putative HRE sites (HRE-A, HRE-B and HRE-C) were identified in the promoter region of the BCL9 gene. (Figure 4A) To study whether these putative HREs were responsible for the hypoxia-mediated induction of BCL9, the DNA fragments containing one copy of these putative HRE sites were inserted into a pGL3 luciferase reporter plasmid. Knowing that VEGF is a hypoxia-inducible gene, we inserted the DNA fragment containing the HRE in VEGF promoter into a pGL3 luciferase reporter plasmid to serve as a positive control. HepG2 and smmc-7721 cells were transiently transfected with these reporter constructs, and then exposed to hypoxia for 36 h. TK100 plasmids were co-transfected as an internal standard to normalize transfection efficiency. The results showed that hypoxia significantly increased the luciferase activity of VEGF promoter, the HRE-B and HRE-C from the BCL9 promoter, while HRE-A from the BCL9 promoter did not respond to hypoxia in both HepG2 and smmc-7721 cells. (Figure 4B) These results demonstrate that hypoxia transactivated the BCL9 promoter containing functional HRE, including HRE-B and HRE-C.

To further investigate which HIF-α (HIF1α or HIF2α) mediated the hypoxia-induced BCL9 expression, chromatin immunoprecipitation (ChIP) assays were applied to determine whether HIF1α and HIF2α physically bound to HREs, especially HRE-B and HRE-C in the BCL9 promoter. HepG2 cells were cultured under the normoxic or hypoxic condition for 36 h, and ChIP assays were undertaken by using antibodies against HIF1α or HIF2α. It was found that HIF1a, but not HIF2a, bound to the HRE-B and HRE-C region in the BCL9 promoter, using VEGF as the positive control. (Figure 4C) The specific pull-down of chromatin fragment containing HRE-B and HRE-C but not HRE-A by the HIF1α antibody under the hypoxic condition was further confirmed by conventional PCR followed by agarose gel electrophoresis (Fig. 4D). It was found that HIF1α rather than HIF2α bound to HRE-B and HRE-C in the BCL9 promoter under the hypoxic condition, which further mediated the hypoxia-induced BCL9 expression. To directly confirm that it was HIF1α that transcriptionally induced the BCL9 expression, HepG2 cells were transfected with the plasmids expressing HIF1α (pcDNA3-Flag-HIF1α). The ectopic expression of HIF1α clearly increased the expression of BCL9 at protein levels as determined by Western-blot assays. (Figure 4E) Furthermore, HIF1a could also bind to the HRE-B and HRE-C region of the BCL9 promoter. (Figure 4F) These results further confirmed that HIF1α interacted with HRE-B and HRE-C in the BCL9 promoter, which is consistent with the ChIP result obtained in cells under the hypoxic condition. (Figure 4G) Collectively, these results suggest that HIF1α rather than HIF2α interacted with HRE-B and HRE-C in the BCL9 promoter to directly induce BCL9 expression under the hypoxic condition.

### Construction of stable knockout cell lines using TALENs

**Figure 5:**
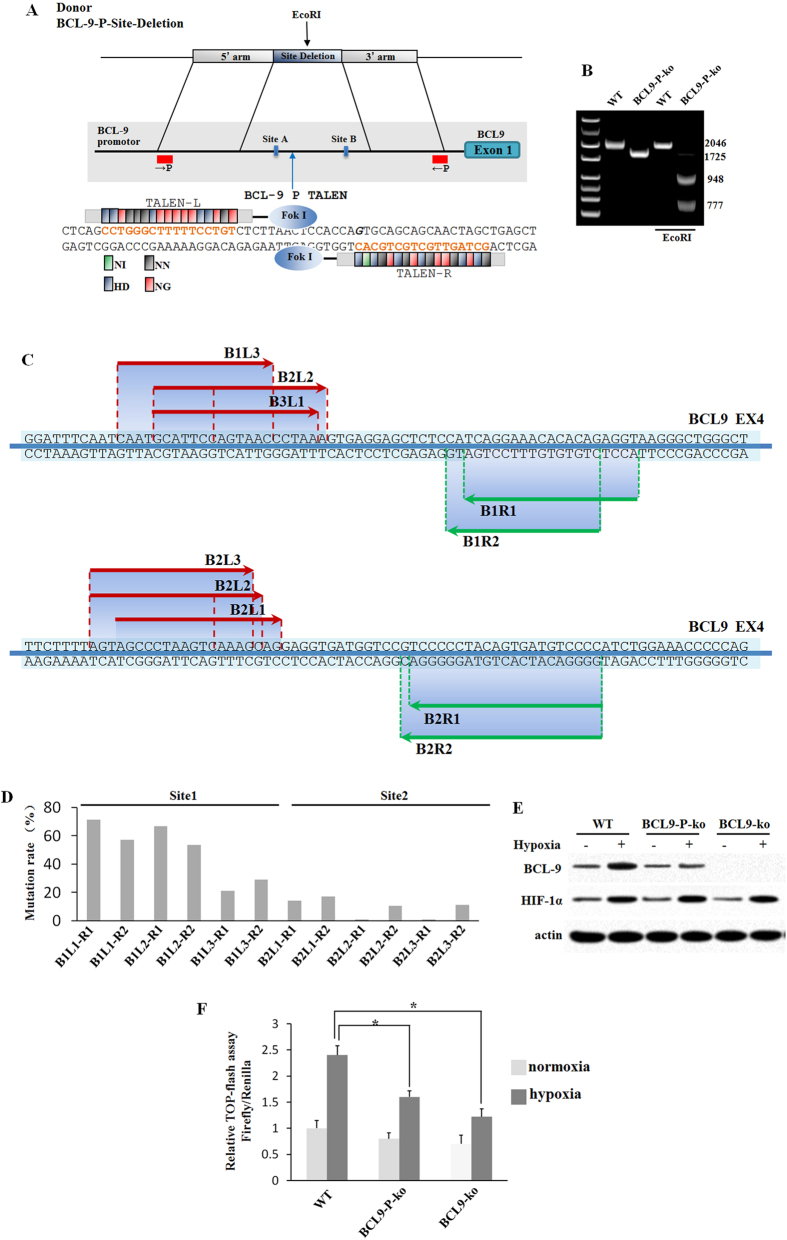
Genetic engineering of HepG2 cells using TALENs. (**A**) Schematic overview depicting the targeting strategy for BCL9 promoter. Primers are shown as red boxes; the blue arrow indicates the cut site by the TALENs. Donor plasmids: CMV promoter, human cytomegalovirus (CMV) immediate early promoter gene; eGFP, enhanced green fluorescent protein gene; Below, scheme of BCL9 TALENs and their recognition sequence. TALE repeat domains are colored to indicate the identity of the repeat variable diresidue (RVD); each RVD is related to the cognate targeted DNA base by the following code (N1 = A, HD = C, NN = G, NG = T). **(B)** Genomic PCR and restriction digestion characterization of BCL9-p-ko HepG2 cells. **(C)** Different TALEN pairs were designed. **(D)** The activities of each two TALEN constructs were examined by SSA assay, and construct L1-R1 showed the highest activity among the constructs in the assay. **(E)** The genomic sequences around the target site of the clones were detected. **(F)** BCL9-wt, BCL9-p-ko and BCL9-ko HepG2 cells were cultured under the hypoxic condition. The BCL9 and HIF-1α protein levels were determined by Western-blot assays. Data are presented as mean ± SD (n = 3). *p < 0.01, Student’s *t*-test.

To gain a more comprehensive understanding of the regulatory role of BCL9 in the development of HCC, we established stable BCL9 promoter knockout (BCL9-p-ko) and BCL9 gene knockout (BCL9-ko) HepG2 cell lines by using TALENs. The schematic overview of the targeting strategy for BCL9 promoter is depicted in Fig. 5A. HepG2 cells were co-transfected with the TALEN pairs targeting the BCL9 promoter region along with Donor plasmids. After G418 treatment, cell clones were expanded and identified by genome PCR and sequencing. (Figure 5B) The plasmids of TALENs for BCL9 gene were designed and constructed using Fast TALE^TM^ TALEN Assembly kit from SiDanSai biotechnology (SiDanSai, China). Three left TALEN plasmids and three right TALEN plasmids were constructed and the target sites are shown in Fig. 5C. The activities of each two TALEN constructs were examined by SSA assay, and construct L1-R1 showed the highest activity among the constructs in the assay (Fig. 5D). Then, construct L1-R1 was introduced into HepG2 by lipofection. After 14-day culture, 50 clones were obtained and the genomic sequences around the target site of the clones were detected. The BCL9-p-ko, BCL9-ko and wild type HepG2 cell lines were treated by hypoxia (0.1% O_2_). The induction of BCL9 expression by hypoxia was confirmed at the protein levels by using Western-blot assay (Fig. 5E). The TOPflash experiments were performed by using WT, BCL-9-ko and BCL9-ko cells. The results were similar to those obtained by siRNA. (Figure 5F).

### BCL9 promotes tumor growth *in vivo*

**Figure 6:**
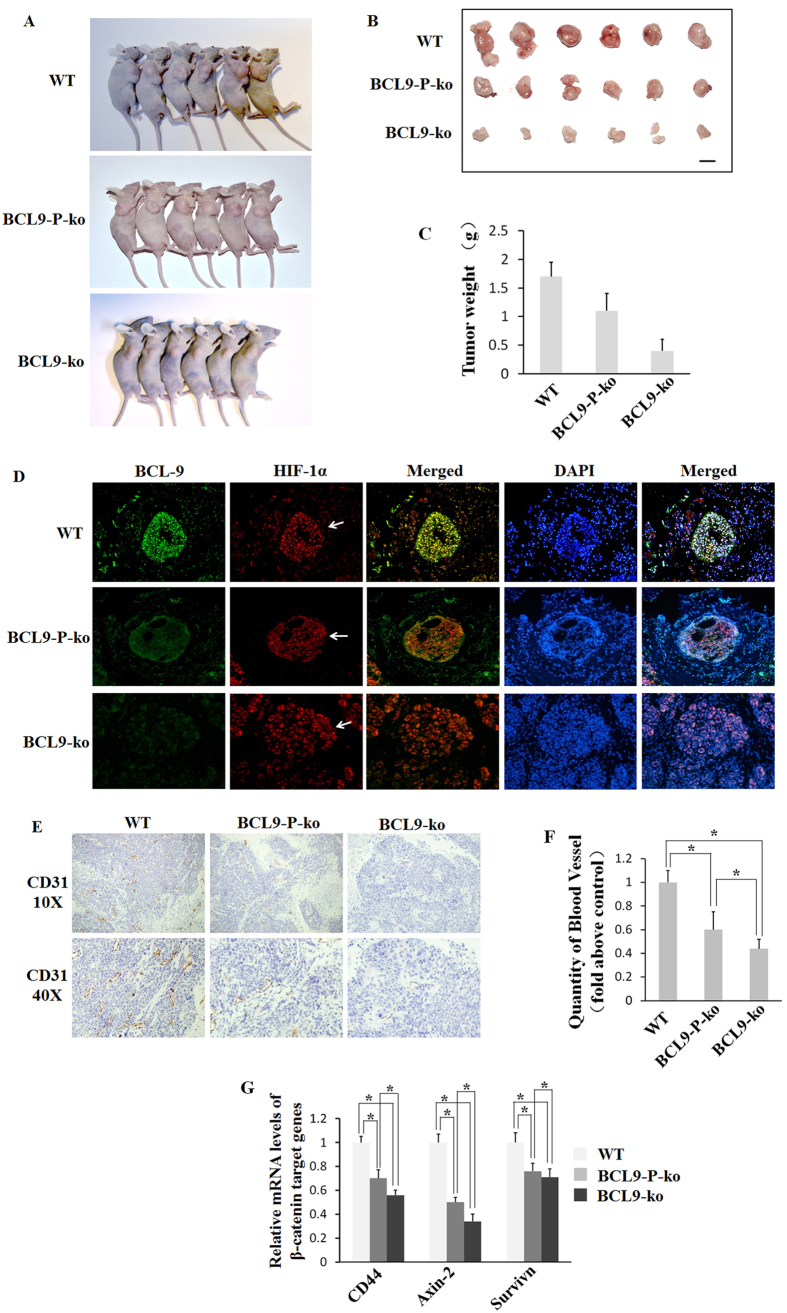
BCL9 promotes the tumor growth *in vivo*. (**A**) The HepG2 cells infected with BCL9-wt, BCL9-p-ko and BCL9-ko were injected subcutaneously into the nude mice. **(B)** Mice were sacrificed to remove the tumors, and images were taken with a Nikon camera. **(C)** Tumor weight was evaluate among BCL9-wt, BCL9-p-ko and BCL9-ko group. **(D)** HIF-1α (red) and BCL9 (green) detected by FISH and IF in tumors: HIF-1α normally expressed in BCL9-wt, BCL9-p-ko and BCL9-ko groups; BCL9 normally expressed in BCL9-wt, weakly expressed in BCL9-p-ko group and unexpressed in BCL9-ko group. **(E,F)** Angiogenesis was detected by IHC and RT-PCR: angiogenesis was significantly decreased in BCL9-p-ko and BCL9-ko group. **(G)** WNT/β-catenin target genes, including CD44, Axin-2 and Survivin, were detected by RT-PCR. CD44, Axin-2 and Survivin were significantly decreased in BCL9-p-ko and BCL9-ko group. Data are presented as mean ± SD (*n* = 3). *p < 0.01, Student’s *t*-test.

To further evaluate the role of BCL9 in the progression of human HCC, we established a xenograft tumor model. Eighteen male nude mice aged 6 weeks were equally randomized to 3 groups. 2 × 10^6^ HepG2 cells transfected with BCL9-wt, BCL9-p-ko and BCL9-ko were injected subcutaneously into the right forearm. Tumor formation was monitored weekly after initiation of tumor cell implantation (Fig. 6A). Mice were euthanized and tumors were removed for further analysis 6 weeks after implantation. The tumor volume and weight were obviously decreased in BCL9-p-ko and BCL9-ko groups. (Figure 6B and C) HIF1α (red) and BCL9 (green) were detected by immunofluorescence. It was found that HIF1α was normally expressed in BCL9-wt, BCL9-p-ko and BCL9-ko groups, and that BCL9 was normally expressed in BCL9-wt, weakly expressed in BCL9-p-ko group, and unexpressed in BCL9-ko group (Fig. 6D). Angiogenesis was detected by immunohistochemistry and RT-PCR. It was found that angiogenesis was significantly decreased in BCL9-p-ko and BCL9-ko groups (*P* < 0.01) (Fig. 6E and F). WNT/β-catenin target genes, including CD44, Axin-2 and Survivin, were detected by RT-PCR. It was found that CD44, Axin-2 and Survivin were significantly decreased in BCL9-p-ko and BCL9-ko groups (*P* < 0.01). (Figure 6G).

### HIF-1α overexpression is associated with BCL9 overexpression in human HCC specimens

**Figure 7:**
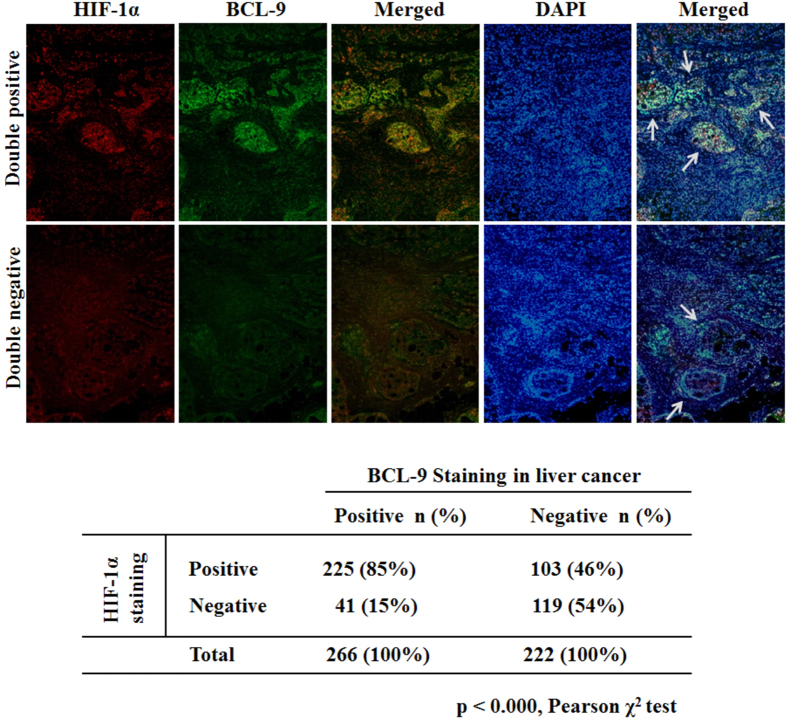
HIF-1α overexpression is associated with BCL9 overexpression in human HCC. HIF-1α and BCL9 protein levels were detected by IHC staining in tissue microarrays containing 488 cases of human HCC specimens. **(A)** HIF-1α (red) and BCL9 (green) detected by FISH and IF in human HCC specimens. **(B)** HIF-1α overexpression is associated with BCL9 overexpression in human HCC (*p* < 0.000, Fisher exact test).

Knowing that hypoxia-induced BCL9 plays an essential role in the progression of HCC *in vitro* and vivo, we examined whether the expression of HIF1α and BCL9 was correlated with the human HCC specimen using double immunofluorescence co-localization. Altogether 488 human HCC specimens were examined. The typical immunofluorescence images of BCL9 and HIF1α are shown in Fig. 7A. 54.5% human HCC specimens showed positive BCL9 staining, and 67.2% showed positive HIF1α staining. Notably, HIF-1α overexpression was significantly correlated with BCL9 over-expression (Fig. 7B) (P ＜ 0.000, Fisher exact test). HIF-1α positive staining was observed in 84.6% (225/266) of the cases with BCL9 positive staining *vs.* 46.4% (103/222) of the cases with BCL9 negative staining. These results strongly suggest that the transcriptional induction of BCL9 by hypoxia and HIF-1α is an important mechanism accounting for the BCL9 overexpression in human HCC specimens.

## Methods

### Clinical samples

Primary HCC, bone metastatic and normal liver tissue specimens were collected from patients who underwent surgical treatment at the Eastern Hepatobiliary Surgery Hospital and Changzheng Hospital of the Second Military Medical University (Shanghai, China) between 2000 and 2013. Tumor tissue specimens were formalin fixed and paraffin embedded. There were 360 human HCC, 72 bone metastatic and 30 normal liver tissue specimens. The age of the patients ranged from 18 to 80 years with a mean of 47.53 ± 12.13 years. Primary HCC differentiation was graded histologically according to the criteria of Edmondson and Steiner^31^. The research protocol was approved by the Ethics Committee of the Second Military Medical University, and written informed consent was obtained from all participants. All methods were performed in accordance with the relevant guidelines.

### Immunohistochemical and immunofluorescence staining

Immunnohistochemical staining for BCL9 was performed by using the standard histological procedure described in the manual for Histostain-Plus IHC Kit, DAB (Phygene). BCL9 staining was scored according to two investigators with previous protocols as negative (−), weak positive (+), and strong (++). The results were analyzed by standard light microscopy. Scoring was assessed blindly with respect to the histologic grade of HCC specimens. Immunofluorescence staining was performed with primary and secondary antibodies diluted in 10% BSA, and the nucleus was stained by DAPI (Sigma). The antibody against HIF-1α (sc-13515, Santa Cruz Biotechnology, 1:500), antibody against HIF-2α (sc-13596, Santa Cruz Biotechnology, 1:500), antibody against BCL9 (ab37305, Abcam, 1:500 dilution) and antibody against β-actin (A5441, Sigma) were used in this study. The antibodies are anti-rabbit in this study. All fluorescent secondary antibodies were used at a dilution of 1:200 for 30 min (invitrogen). Quantification of immunofluorescence was performed using NIH Image J.

### Cell lines and culture conditions

Human HCC HepG2 and smmc-7721 cells were obtained from the American Type Culture Collection (ATCC), and maintained in DMEM supplemented with 10% fetal bovine serum (Gibico) in the cell incubator (37 °C, 5% CO2). For hypoxic treatment, cells at 50–60% confluence were incubated in a hypoxia chamber (0.1% O_2_, STEMCELL).

### Cell proliferation, migration and angiogenesis

BCL9 expression plasmids (pcDNA3-Flag-BCL9) and HIF1α (pcDNA3-Flag-HIF1α) were purchased from GENEwiz^TM^. Two different siRNA oligos against BCL9 were obtained from Ribobio^TM^ for siRNA knockdown. SiRNA targeting BCL9: siRNA-1: 5-CAG ACU UUA UGU UCA UAG UUC UUC CUC-3; siRNA-2: 5-CUU CCA CAA CUA CAU AGG GUA UUG UUU-3. Expression plasmids and siRNA oligos were transfected into cells using X-tremeGENE HP (Roche).

MTS assay was used for cell viability analysis according to the manufacturer’s instructions (Promega, Fitchburg, WI, USA). The results were expressed as viability indices representing relative percentages compared to the controls. For migration assay, HepG2 and smmc-7721 cells were seeded in the upper chamber of Transwell plates (BD Bioscience) without or with Matrigel, under serum-free conditions. Medium supplemented with 10% fetal calf serum (FCS) and 50% ug/mL fibronectin (BD Bioscience) was used as a chemoattractant in the lower chamber. After 10-h incubation, cells remaining in the upper chamber were removed with a cotton swab, while cells adhering to the lower membrane were stained with 0.1% crystal violet and photographed with an inverted microscope (Zeiss). The area of positive staining was measured using image analysis software (Image-Pro Plus 6.0, Media Cybernetics). Migration was calculated as the positive area percentages. For angiogenesis analysis, 50 μl chilled Matrigel (BD Bioscience, Franklin Lakes, NJ, USA) was added to a 96-well plate and incubated at 37 °C for 30 min. HUVECs (1 × 104 cells) were suspended in 100 μl EBM-2 and conditioning medium from each treated cells, and added to the solidified Matrigel. After 18-h incubation, angiogenesis was assessed on the basis of capillary-like structure formation. Tubes in randomly chosen five microscopic fields were photographed and counted. At least three independent experiments were performed for each condition.

### RT- PCR

Total RNA was isolated by using RNAiso Plus (TAKARA) following the manufacturer’s instruction. RNA was reverse transcribed into cDNA by using the Taqman Reverse Transcription Reagents kit (Applied Biosystems) with random hexamers. Human BCL9, VEGF, HIF1α, HIF2α, actin, survivin, CD44 and axin 2 mRNA levels were determined in 7900HT Fast Real-time PCR system (Life Technology Corporation, USA). All primers were purchased from Applied Biosystems. RT-PCR was done in triplicate with TaqMan PCR mixture (Applied Biosystems). The expression of genes was normalized to the actin gene.

### Plasmids

The fragments containing the potential HRE sites (5′-A/GCGTG-3′) identified from the BCL9 promoter (A: −4087 bp ~ −3016 bp; B: −1078 bp ~ −323 bp; C: −313 bp ~ 822 bp) and VEGF promoter were amplified by following PCR primers. For HRE-A, forward primer: 5′-AGA TGG TCT CCG TCT CCT-3′, reverse primer: 5′-GAG CCT TGC TAT CTG AAC-3′; for HRE-B, forward primer: 5′-CTT ATC TCC CTA CTC CCC-3′, reverse primer: 5′-GAG ACA GGA AAA AGC CCA-3′; for HRE-C, forward primer: 5′-CAG TGC AGC AGC AAC TAG-3′, reverse primer: 5′-GAC AAG CCA CAA ACA AGA C-3′; for VEGF promoter, forward primer: 5′- CCT CAG TTC CCT GGC AAC ATC TG-3′, reverse primer: 5′-GAA GAA TTT GGC ACC AAG TTT G-3′. For KLF4, forward primer: 5′- AGC AAA GGC AAT TGG AGA GA-3′, reverse primer: 5′-GAG TCC AAG GCA GTT CGT GT-3′. Each of the forward primers and reverse primers contained a KonI site and an XhoI site, respectively. These PCR fragments were cloned into pGL3-Basic Luciferase Reporter Vector (Promega) at KpnI-XhoI restriction sites. All constructs were confirmed by DNA sequencing.

### Luciferase reporter assay

To test whether hypoxia activated the WNT/β-catenin pathway, HepG2 and smmc-7721 cells were transfected with DNA plasmids of β-catenin-LEF/TCF-sensitive (TOP-flash) or β-catenin-LEF/TCF insensitive (FOP-flash) reporter vectors (Addgene, Cambridge, MA). The following amounts of plasmids were used per well of a 24-well plate: 1 μg and 0.5 μg of TOPflash as well as 1 μg and 0.5 μg of FOPflash for HepG2 and smmc-7721 cells, respectively. To test whether hypoxia transactivated the pGL3-reporter plasmids described above, HepG2 and smmc-7721 cells were transfected with the pGL3-reporter plasmids containing one copy of each potential HRE site. To further test the transactivation activity of HIF1α on pGL3-reporter plasmids, cells were co-transfected with pGL3-reporter plasmids together with HIF1α expression plasmids. The X-tremeGENE HP (Roche) transfection agent was used according to the manufacturer’s instructions. phRL-TK plasmid (Promega, Madison, WI) was co-transfected as internal controls. Forty-eight hours after transfection, cells were then treated by hypoxia for 48 h. The luciferase activity was measured using the Dual Luciferase assay kit (Promega) and normalized with the internal standard. At least three independent experiments were carried out for each condition.

### ChIP assay

ChIP assay was performed by using a ChIP assay kit (Millipore) in accordance with the manufacturer’s instructions. HepG2 and smmc-7721 cells treated with hypoxic or normoxic conditions or transfected with HIF1α expression plasmids were lysed in 150ul SDS buffer. Chromatins in these cells were then cross-linked with 1% formaldehyde for 10 min at room temperature, and then sonicated to generate 200 to 500 bp DNA fragments in lysis buffer. The DNA fragments were subjected to ChIP assays with anti-HIF-1α or anti-HIF2α antibodies. Normal IgG was used as a control for nonspecific binding of genomic DNA. Also, we employed transcription factor KLF4 to regulate WNT5A gene as control, knowing that it is not affected by hypoxia^32^. The enrichment of the eluted DNA was assessed by RT-PCR and conventional PCR by using the PCR primer sets described above with the omission of restriction enzyme recognition sequence.

### Construction of stable knockout cell lines using TALENs

The schematic overview of the targeting strategy for BCL9 promoter is depicted in Fig. 5A. HepG2 cells were co-transfected with the TALEN pairs targeting the BCL9 promoter region along with Donor plasmids. After G418 treatment, cell clones were expanded and identified by genome PCR and sequencing. The plasmids of TALENs for BCL9 gene were designed and constructed using Fast TALETM TALEN Assembly kit from SiDanSai biotechnology (SiDanSai, China). The constructed TALEN constructs were transfected into 293 T cells. To validate the activities of synthesized endonucleases, an equal amount of expression vectors for the left and right TALENs or sgRNA were transferred into HEK293 cells, and T7E1 mismatch-specific nuclease assay was performed. The TALENs for BCL9 gene were transfected into HepG2. After selection with puromycin, resistant colonies were picked up and examined by genomic PCR and gene sequencing. Clones with successful knockout of BCL9 promotor and BCL9 gene were selected for the xenografts.

### Animals

Animal use was according to the National Institutes of Health (NIH) guidelines, and the care and treatment of all animals were in strict accordance with protocols approved by the Institutional Animal Experiment Committees of the Second Military Medical University. Eighteen male nude mice aged 6 weeks were equally randomized to 3 groups. To detect the effect of BCL9 on the tumorigenicity of HCC cells, 2 × 10^6^ HepG2 cells transfected with BCL9-p-ko, BCL9-ko and BCL9-wt were injected subcutaneously into the right forearm of the animal. Tumor formation was monitored weekly after initiation of implantation. Mice were euthanized and tumors were removed for further analysis 6 weeks after implantation. The tumor volume and weight were estimated. BCL9 and HIF-1α level and angiogenesis were detected. The β-catenin pathway targeted genes, including CD44, Actin-2 and Survivin expression were also measured^33^,^34^,^35^.

### Statistical analysis

The data were expressed as mean ± SD. The association between HIF-1α expression levels and BCL9 expression levels was analyzed by Fisher exact test. All other *p* values were obtained by using two-tailed Student’s *t*-test. Values of *p* < 0.05 were considered to be statistically significant.

## Discussion

The canonical Wnt/ β-catenin signaling pathway is known to be involved in the pathogenesis of a wide variety of cancers. Nuclear β-catenin plays a key role in this pathway by functioning as a transcription factor to activate the expression of cell proliferation, migration, and survival genes such as c-MYC and CyclinD1^6^,^7^. BCL9 is an essential co-activator in the Wnt/β-catenin signaling pathway by mediating the recruitment of pygopus to the nuclear β-catenin-TCF complex^8^. Despite the mutational status of the Wnt signaling components, BCL9 could also enhance β-catenin-mediated transcription activity and increase cell proliferation, invasion and migration^12^. BCL9 is absent in normal cells and frequently over-expressed in a variety of solid tumors including colorectal cancers, multiple myeloma and HCC^12^. Importantly, the over-expression of BCL9 in tumors is associated with poor prognosis of HCC patients^13^. There is evidence that BCL9 is a bona fide oncogene^12^,^14^,^15^. However, the mechanism underlying the effect of BCL9 over-expression in tumors remains unclear.

It was found in this study that BCL9 was overexpressed in HCC specimens, especially in bone metastasis specimens. BCL9 was reported to be an important factor in determining the proliferation, migration, invasion and the metastatic potential of multiple myeloma and colon carcinoma cells^12^. In this study, we also found that BCL9 promoted cell proliferation, migration and angiogenesis by overexpressing or knocking down BCL9 in HepG2 and smmc-7721 cell lines. These data together indicate that BCL9 plays a role in the progression and metastasis of human HCC.

Previous studies^15^,^36^ showed reduced BCL9 mRNA and protein expressions upon ectopic expression of miR-30s, suggesting that the expression of BCL9 was regulated by miR-30s post-tanscriptionally. However, the mechanism for BCL9 transcription in tumors has not been reported. Here we provided evidence for the first time that hypoxia, which is very common in solid tumors, increased the expression of BCL9 in human HCC cell lines. Cellular responses to hypoxia are mediated by HIFs. In humans, HIFs are heterodimeric transcription factors consisting of HIF-α and HIF-β that are expressed constitutively at the transcriptional and translational levels. Once activated under hypoxia, HIFs bind to DNA containing a HRE (5′-G/ACGTG-3′)^37^. HIF-1α and HIF-2α are the two best-studied members of the HIF-α family^38^. HIF-1α and HIF-2α proteins can form dimers with HIF-β and bind to HRE to effectively regulate the expression of their target genes^39^. Although HIF-1α and HIF-2α induce a number of common genes, and at the same time they have their respective unique target genes^40^,^41^. In addition, the difference in HIF1α and HIF2α target gene selection can be attributed to their interactions with different transcription factors and/or chromatin context^42^,^43^. Therefore, experiments elaborating the precise roles of each subunit are needed^19^,^44^. In the current study, we found that BCL9 was a target gene of HIF-1α that interacted with HRE-B and HRE-C but not HRE-A in the BCL9 promoter under the hypoxic condition. Of relevance the expression of BCL9 and HIF1α transcripts was significantly correlated, because both transcripts were highly expressed in HCC specimens. To the best of our knowledge, it is the first time to demonstrate that HIF1α rather than HIF2α interacted with HRE-B and HRE-C in the BCL9 promoter to directly induce BCL9 expression under the hypoxic condition.

The hypoxia signaling pathway is known to regulate multiple steps of tumorigenesis and related to poor clinical outcomes^45^. Due to restricted blood flow, solid tumor cells may experience hypoxia, which reduces cell proliferation or death, or even energy metabolism from the oxidative phosphorylation pathway to the glycolysis pathway as a response to hypoxia, resulting in modifications of hypoxia-inducible genes expression and treatment outcome in cancer patients^46^. HCC was also reported to display hypoxia, which could be a potential therapeutic target^47^. It was reported that the Wnt/β-catenin signaling pathway was activated at least 95% HCC patients^48^. Several studies^26^,^49^. reported the crosstalk between Wnt/β-catenin and hypoxia signaling pathway, but the mechanism has not well documented due to the complexity of the network between Wnt/β-catenin and hypoxia signaling pathway. In this study, we for the first time provided the evidence of crosstalk between canonical Wnt/β-catenin and hypoxia signaling in that hypoxia led to the upregulation of the key β-catenin coactivator BCL9 in a HIF1α-dependent manner, suggesting that the hypoxia pathway might promote oncogenic Wnt/β-catenin signaling by recruiting HIF-α/BCL9 interaction. These findings may enlarge our views on the interaction of Wnt/β-catenin and hypoxia signaling pathways.

In conclusion, this study demonstrated that BCL9 promoted cell proliferation, migration and angiogenesis in human HCC cell lines. BCL9 expression could be stimulated by hypoxia. BCL9 induction under the hypoxic condition was predominantly mediated by HIF1α. Due to the critical role of BCL9 in promoting tumor development in the Wnt/β-catenin signaling pathway, the specific regulation of BCL9 expression by HIF1α is considered to be an underlying crosstalk between Wnt/β-catenin signaling and hypoxia signaling pathways. These results could serve as a foundation for study of new targets for development of potential therapeutic strategies by blocking HIF-1α/BCL9 signaling related overexpression in human HCC.

## Additional Information

**How to cite this article**: Xu, W. *et al*. Hypoxia activates Wnt/β-catenin signaling by regulating the expression of BCL9 in human hepatocellular carcinoma. *Sci. Rep.* **7**, 40446; doi: 10.1038/srep40446 (2017).

**Publisher's note:** Springer Nature remains neutral with regard to jurisdictional claims in published maps and institutional affiliations.

## Supplementary Material

Supplementary Material

## Figures and Tables

**Table 1:** Comparison of BCL9 expression level in specimens from normal liver tissue, primary hepatocellular carcinoma and bone metastasis.

Viable	Specimen			*P*-value
	Normal liver tissue n = 30	Primary HCC n = 360	Bone metastasis n = 72	
Age	44.30 ± 10.5	47.17 ± 12.60	50.64 ± 9.68	—
Gender				
Male	23	298	58	0.66
Female	7	62	14	
BCL9 expression				
Negative (−)	30	236	7	0.00
Moderate (+)	0	93	24	
Strong (++)	0	31	41	
