# Supplementary material for: Hypoxia activates Wnt/β-catenin signaling by regulating the expression of BCL9 in human hepatocellular carcinoma
Source: Sci Rep. 2017 Jan 11;7:40446. doi: 10.1038/srep40446 (PMC5225427; doi:10.1038/srep40446)
Supplement: Supplementary Material [file srep40446-s1.pdf]

Hypoxia activates Wnt/β-catenin signaling by regulating the expression of BCL9 in human hepatocellular carcinoma

Wei Xu, MD[1*]; Wang Zhou, MD[1*]; Mo Cheng, MD[1*]; Jing Wang, MD[1,3*]; Zhian Liu, MD[3]; Shaohui He, MD[1]; Xiangji Luo, MD[4]; Wending Huang, MD[2]; Tianrui Chen, MD[1]; Wangjun Yan, MD[1,2Δ] and Jianru Xiao, MD[1Δ]

1. Department of Orthorpedic Oncology, Changzheng Hospital, Second Military Medical University, NO. 415, Fengyang Road, Shanghai, 200003, China.

2. Department of Bone and soft tissue tumors, Fudan Cancer Center, Fudan University, NO. 270, Dong'an Road, Shanghai, 200000, China

3. Department of Anatomy, Xuzhou Medical University, NO. 209, Tongshan Road, Xuzhou, 221004, China

4. Department of Biliary Surgery, Eastern Hepatobiliary Surgery Hospital, Second Military Medical University, NO. 225, Changhai Road, Shanghai, 200438, China

[*] These authors contributed equally to this study.

[Δ]Corresponding author:

Jianru Xiao and Wangjun Yan, Department of Orthorpedic Oncology, Changzheng Hospital, Second Military Medical University, 415 Fengyang Road, Shanghai, 200003, China. Phone:(086)021-13701785283, Fax: (086)021-81885634, Email: jianruxiao83@163.com and 13917966770@163.com

Disclosures:

Conflicts of interest: The authors disclose no conflicts.

Supplementary table 1. Correlation of BCL9 expression and clinicopathological parameters in hepatocellular carcinoma patients (N = 360)

**Variable**	**n**	**BCL9 expression**			***P*-value**
		-	+	++	
**Age**					
**≤55**	260	162	72	26	0.09
**>55**	100	74	21	5	
**Gender**					
**Male**	298	192	83	23	0.10
**Female**	62	44	10	8	
**Tumor size (cm)**					
**≤5.0**	133	89	33	11	0.92
**> 5.0**	227	147	60	20	
**Edmondson**					
**invasion**	81	66	15	0	**0.00**
**I**	242	162	64	16	
**II**	37	8	14	15	
**III**					
**Microvascular invasion**					
**(-)**	31	28	3	0	0.01
**(+)**	329	208	90	31	
**T-Stage**					
**1**	32	28	4	0	**0.02**
**2**	195	119	56	20	
**3**	105	74	21	10	
**4**	28	15	12	1	
**Lymphnode metastasis**					
**(-)**	332	218	83	31	0.15
**(+)**	28	18	10	0	
**Metastasis**					
**(-)**	357	235	91	31	0.26
**(+)**	3	1	2	0	

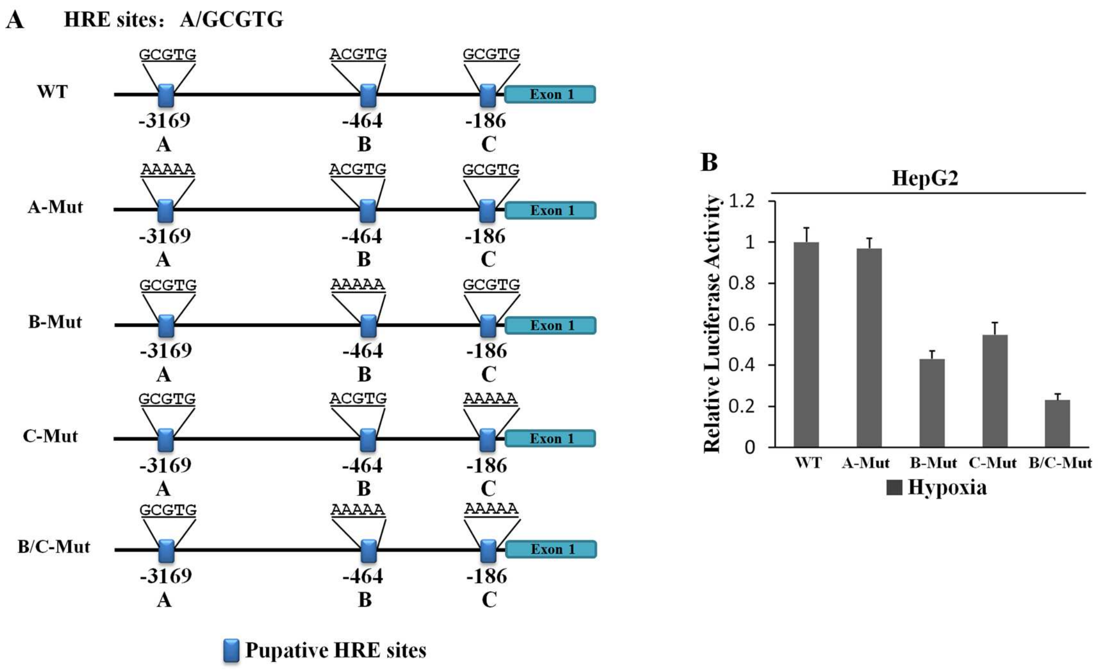

Supplementary Figure 1. Mutating the HRE site confirmed that hypoxia interacted with HRE-B and HRE-C in the BCL9 promoter to induced its expression. (A) The HRE-A mutant (A-Mut), HRE-B mutant (B-Mut), HRE-C mutant (C-Mut) as well as HRE-B/C mutant (B/C-Mut) were constructed, respectively. **(B)** The luciferase activities were significantly suppressed in B-Mut, C-Mut and B/C-Mut groups after treatment of hypoxia.
